# Longitudinal clinical outcomes after amyloid clearance in aducanumab phase 3 studies

**DOI:** 10.1002/alz.71758

**Published:** 2026-10-07

**Authors:** Menglan Pang, Holly M. Brothers, John O'Gorman, Jennifer Murphy, Yuval Zabar, Tyler Lasky, Changyu Shen, Ivana Rubino, David Knopman

**Affiliations:** ^1^ Biogen Inc. Cambridge Massachusetts USA; ^2^ Department of Neurology Mayo Clinic Rochester Minnesota USA

**Keywords:** Alzheimer's disease, amyloid negative, anti‐amyloid antibody, anti‐amyloid therapy, mild cognitive impairment, treatment‐related amyloid clearance

## Abstract

**INTRODUCTION:**

Anti‐amyloid antibody clinical trials with a high proportion of participants reaching treatment‐related amyloid clearance (TRAC) had positive clinical outcomes. We evaluated the aducanumab phase 3 program to determine whether TRAC during the double‐blind period was likewise associated with clinical benefit.

**METHODS:**

We pooled ENGAGE and EMERGE (E/E) aducanumab‐treated participants in the amyloid positron emission tomography substudy who continued into the long‐term extension ≤ 134 weeks (continuously treated population) and/or returned to the EMBARK redosing study 0.7 to 2.5 years after termination of E/E (treatment gap population). Clinical outcomes were stratified by week 78 TRAC status.

**RESULTS:**

TRAC participants had less clinical decline at week 78, week 134, and EMBARK baseline than non‐TRAC participants. Post‐TRAC, clinical decline was heterogeneous; approximately two thirds of TRAC participants had annualized decline of < 1 point on Clinical Dementia Rating Sum of Boxes.

**DISCUSSION:**

In these exploratory analyses, week 78 TRAC status was associated with better clinical outcomes.

**CLINICAL TRIAL REGISTRATION:**

NCT02484547, NCT02477800, NCT04241068

## Highlights

- Long‐term clinical outcomes from the aducanumab phase 3 trials were evaluated.
- Treatment‐related amyloid clearance (TRAC) was defined as amyloid negative at week 78.
- TRAC had less clinical decline than non‐TRAC when treatment was continued.
- TRAC had less clinical decline than non‐TRAC when treatment was stopped.
- Some TRAC patients remained stable while others had meaningful clinical decline.

## 1 BACKGROUND

Alzheimer's disease (AD) is characterized by two distinct protein pathologies: accumulation of extracellular amyloid beta (Aβ) plaques and intracellular tau neurofibrillary tangles. The presence of Aβ plaques is widely considered a necessary, though not solely sufficient, driver of the pathological cascade. The emergence of anti‐amyloid monoclonal antibodies has marked a turning point in the therapeutic landscape, offering the first disease‐modifying treatments to show clinical benefit in large‐scale trials. While overall reduction of amyloid plaque burden is a key therapeutic goal and is correlated with positive outcomes across clinical trials,^1^, ^2^, ^3^, ^4^, ^5^, ^6^, ^7^, ^8^, ^9^ accumulating evidence suggests that the extent of amyloid plaque clearance achieved during treatment may be critical.^5^, ^10^, ^11^ Treatment‐related amyloid clearance (TRAC) is emerging as an important concept in the development and use of anti‐amyloid antibodies because of a potential relationship with clinical outcomes.^10^, ^12^, ^13^

TRAC has been defined as the achievement of amyloid‐negative status via therapeutic intervention resulting in the reduction of amyloid plaque burden as measured by positron emission tomography (PET) below the established diagnostic threshold used to define an elevated state.^12^ An amyloid PET threshold of 19 to 24 Centiloid (CL) has been identified as an inflection point in natural history studies beyond which the rate of change in amyloid PET worsens reliably,^14^, ^15^, ^16^ though levels as low as 15 to 17 CL have been proposed.^17^ Thresholds of 20.2 to 30 CL have been used as inclusion criteria in phase 3 clinical trials of anti‐amyloid antibodies to evaluate amyloid plaque clearance,^1^, ^2^, ^3^, ^4^, ^13^ in which a higher value was chosen to increase certainty of progressive disease. TRAC may be considered amyloid negative, but TRAC does not imply that amyloid plaque has been cleared entirely. TRAC represents a treatment‐induced, modified disease state,^10^, ^12^ and the impact of achieving this clearance on subsequent long‐term clinical outcomes remains a critical knowledge gap.

On a trial‐by‐trial basis, anti‐amyloid antibody programs that achieved TRAC in a high proportion of participants demonstrated clinical benefits,^1^, ^2^, ^3^ while those with lower proportions failed to demonstrate clinical efficacy.^3^, ^4^ These observations have established achievement of TRAC by 52 to 78 weeks of anti‐amyloid therapy as a leading indicator of target engagement. While between‐trial demonstrations are compelling,^5^, ^11^ they lack the validity of demonstrating the benefits of TRAC in a single‐agent trial. An initial evaluation of the aducanumab phase 3 trials suggested that patients who achieved TRAC had better clinical outcomes.^13^

We revisited the pooled aducanumab phase 3 EMERGE and ENGAGE (E/E) amyloid PET substudy dataset^3^ and addressed the relationship between TRAC and clinical outcomes. We first interrogated this dataset to determine which patient characteristics were associated with reaching TRAC after aducanumab treatment. We then stratified participants according to whether they achieved TRAC at week 78 or not in the pooled E/E dataset. We compared TRAC versus non‐TRAC groups who completed the E/E double‐blind trials within two cohorts: (1) the “continuously treated population” comprising participants who entered a long‐term extension (LTE) that was contiguous with the double‐blind trials and extended the observation period to 134 weeks and (2) the “treatment gap population” comprising participants who returned after a treatment‐free gap of 0.7 to 2.5 years and enrolled in the phase 3b EMBARK study in which previously exposed participates were re‐dosed. Finally, we evaluated heterogeneity in clinical outcomes after TRAC and investigated which factors may be associated with the trajectory of disease progression after TRAC.

The LTE and post‐gap observations stratified by TRAC status are unique longitudinal data that inform on two dosing scenarios: continued and stopped. Although the clinical development program was discontinued and aducanumab was later withdrawn from the market for reasons unrelated to safety or efficacy, the insights gained from the extended observations from the phase 3 trials speak to the novel condition of reaching TRAC status after anti‐amyloid antibody therapy. This unique longitudinal data can therefore inform critical aspects of the clinical approach and monitoring strategies for the use of currently commercially available agents, lecanemab^1^ and donanemab.^2^

## 2 METHODS

### 2.1 Clinical trials

All studies (EMERGE [NCT02484547], ENGAGE [NCT02477800], and EMBARK [NCT04241068]) were conducted in accordance with the Declaration of Helsinki and the International Conference on Harmonization Good Clinical Practice guidelines. Ethics committee approval was obtained at each participating site. All participants provided written informed consent.

E/E were randomized, double‐blind, placebo‐controlled, identical phase 3 studies that assessed the efficacy and safety of aducanumab in participants with early AD.^3^ Participants with visually positive amyloid PET scans were randomized 1:1:1 to low dose (3 or 6 mg/kg), high dose (6 or 10 mg/kg), or placebo for 78 weeks. After week 78, participants could enroll in the LTE period for up to 5 years. E/E were terminated early, and patients ended the trial at various times throughout the double‐blind period and LTE.

**RESEARCH IN CONTEXT**

1. **Systematic review**: The authors reviewed the literature using traditional (e.g., PubMed) sources. Trial outcomes in populations in which the majority have achieved amyloid clearance have been described in continuous and stopped dose scenarios. To our knowledge, one clinical program to date has published clinical outcomes stratified by whether participants achieved treatment‐related amyloid clearance (TRAC). There has been related work that investigated clinical outcomes across deciles of lowest post‐baseline amyloid positron emission tomography standardized uptake value ratios. These are cited appropriately.
2. **Interpretation**: These data underscore TRAC as an important factor related to clinical benefit in both continuous and stopped dosing scenarios and illustrate the range of clinical trajectories observed in each after TRAC.
3. **Future directions**: The aducanumab clinical trial program is closed and aducanumab has been withdrawn from market; however, better understanding the impact of removing amyloid plaque pathology on clinical outcomes may inform future research in Alzheimer's disease.

EMBARK was an open‐label, multicenter, longitudinal, single‐arm, global phase 3b study in participants with AD who were actively participating in aducanumab studies at the time that these studies were terminated early (feeder studies included E/E, and two other trials not included in the current analysis: PRIME [NCT01677572] and EVOLVE [NCT02477800]). The primary objective was to evaluate the long‐term safety and tolerability of aducanumab in patients titrated to a monthly dose (10 mg/kg) of aducanumab after a treatment gap period imposed by discontinuation of feeder studies. In 2024, the aducanumab clinical development program was discontinued.

### 2.2 Clinical assessments

The E/E primary endpoint was Clinical Dementia Rating Sum of Boxes (CDR‐SB) at week 78, and secondary endpoints were 13‐item Alzheimer's Disease Assessment Scale Cognitive subscale (ADAS‐Cog13), Alzheimer's Disease Cooperative Study–Activities of Daily Living–Mild Cognitive Impairment (ADCS‐ADL‐MCI), and Mini‐Mental State Examination (MMSE) scores at week 78. Clinical assessments were conducted (Figure 1) throughout the E/E double‐blind period at weeks 26, 50, and 78 and through the E/E LTE at weeks 106, 134, 162, and 182. Clinical assessments were conducted at EMBARK baseline.

**FIGURE 1:**
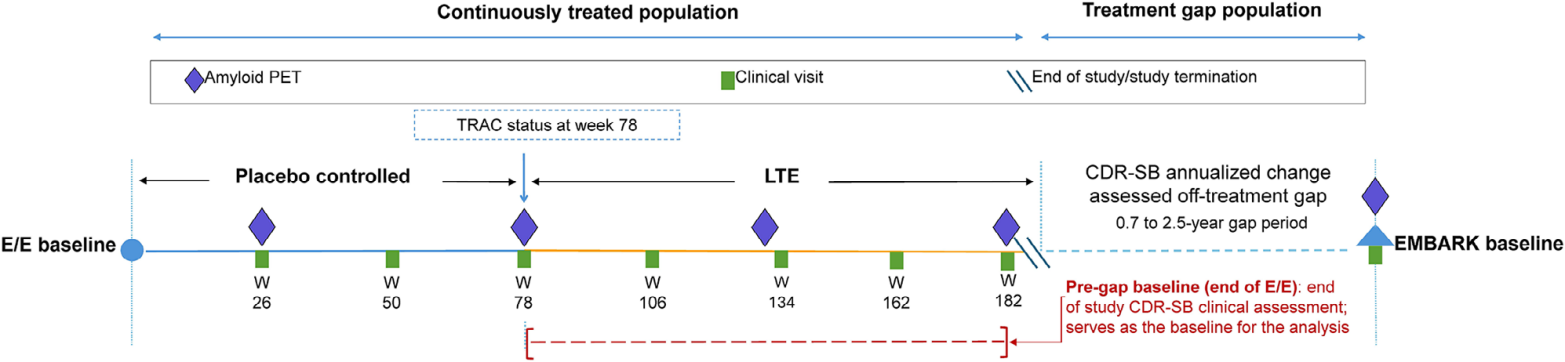
Participants and study design, including timeline of assessments. All participants were from the treated groups of the E/E PET substudy. Some entered the LTE (continuously treated population) and/or the EMBARK redosing study after a variable treatment gap of 0.7 to 2.5 years (treatment gap population). The E/E trials were terminated early (blue hash lines), and the last clinical assessment prior to EMBARK is considered the pre‐gap baseline. Clinical assessments (green squares) were conducted at baseline, throughout the E/E double‐blind period (at weeks 26, 50, and 78), throughout the E/E LTE (at weeks 108, 134, 162, and 182), and at EMBARK baseline. Amyloid PET imaging was conducted at baseline, throughout the double‐blind period (at weeks 26 and 78), throughout the E/E LTE (at weeks 132 and 182), and at EMBARK baseline. Participants were considered to have achieved TRAC if amyloid PET was ≤ 20.2 Centiloid at week 78 (purple diamond). CDR‐SB, Clinical Dementia Rating Sum of Boxes; E/E, EMERGE/ENGAGE; LTE, long‐term extension; PET, positron emission tomography; TRAC, treatment‐related amyloid clearance; W, week.

### 2.3 Amyloid PET substudy and TRAC threshold

A prespecified longitudinal amyloid PET imaging substudy of the E/E trials was conducted. Imaging used ^18^F‐florbetapir or ^18^F‐flutemetomol radiotracers via previously described methods.^3^, ^18^ Participants in the E/E double‐blind period had PET imaging conducted (Figure 1) at weeks 26 and 78. In the LTE period, additional amyloid PET imaging was conducted at weeks 132 and 182.

Positive and negative amyloid PET values were selected for analysis purposes and to aid in consistent interpretation of amyloid PET scans, as previously described.^3^, ^18^, ^19^ The amyloid‐negative PET standardized uptake value ratio (SUVR) value of ≤ 1.1 corresponded to a value of ≤ 20.2 CL at week 78 in the E/E trials^3^ was used as the threshold for TRAC.^3^, ^15^, ^16^, ^17^ This threshold has also been shown to accurately distinguish autopsy‐identified amyloid‐positive cases from amyloid‐negative cases.^20^ The designation of TRAC based on this threshold does not indicate the complete absence of amyloid plaque pathology, nor does it indicate the absence of other forms of amyloid. Participants could reach the amyloid PET threshold after week 78 but would be considered non‐TRAC for the purpose of this analysis.

### 2.4 Key participants and timeline of assessments

Study participants included in the present analyses were from the pooled active‐treatment groups of the E/E PET substudy who had a PET assessment at week 78 and then optionally entered the E/E LTE and/or enrolled in the redosing EMBARK study after a variable treatment gap of 0.7 to 2.5 years (Figure 2).

**FIGURE 2:**
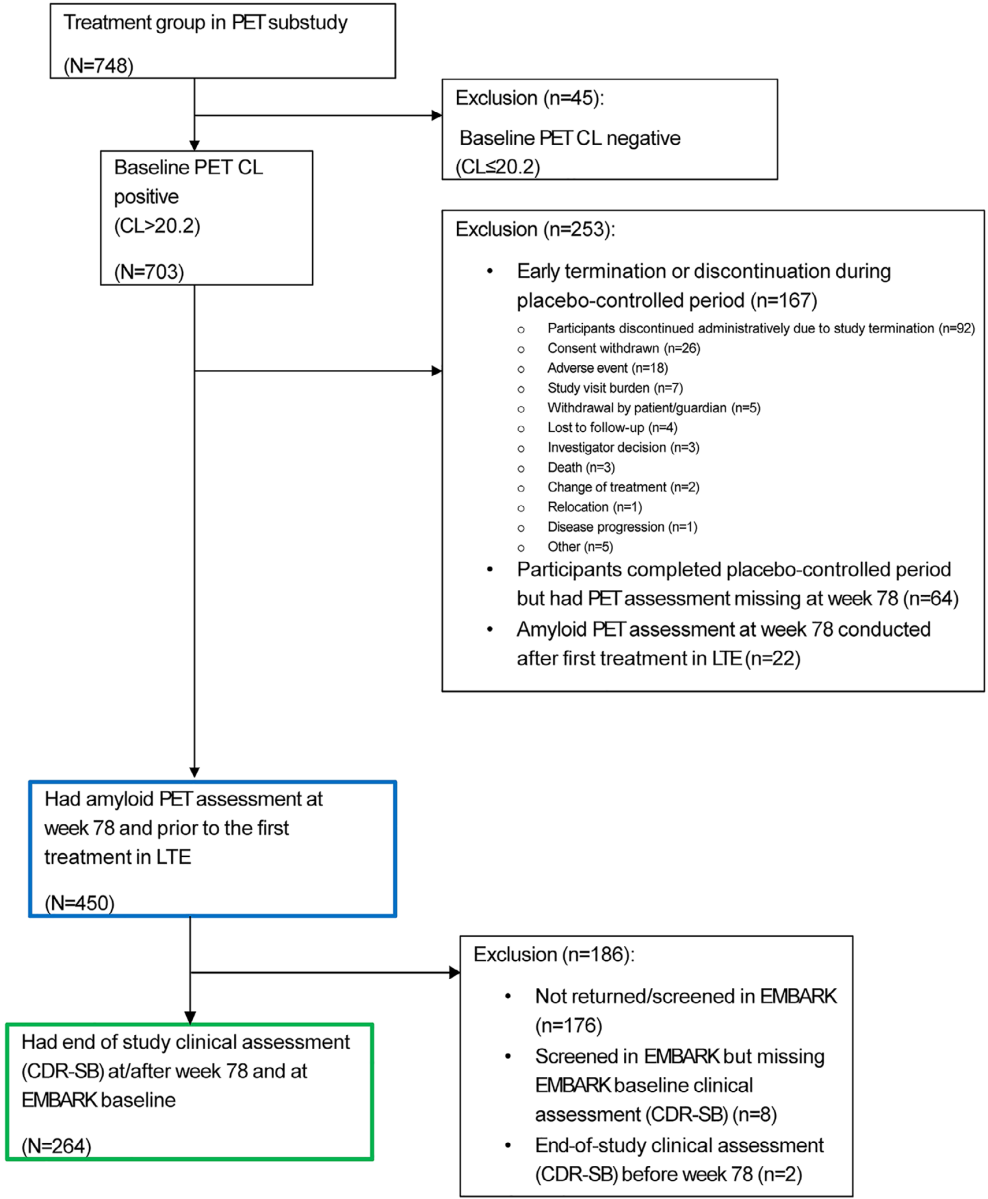
Flowchart of participant selection in the analysis population. The continuously treated population (blue box) included pooled treatment groups of the EMERGE/ENGAGE PET substudy who had amyloid PET > 20.2 CL at baseline and had a PET assessment at week 78 and before the first dose in LTE. The treatment gap population (green box) included participants from the continuously treated population who had a clinical assessment at or after week 78, enrolled in the EMBARK redosing study, and had clinical assessment at EMBARK baseline. The group boxed in blue represents the continuously treated population. The group boxed in green represents the treatment gap population. CDR‐SB, Clinical Dementia Rating Sum of Boxes; CL, Centiloid; LTE, long‐term extension; PET, positron emission tomography.

#### 2.4.1 Analysis population

##### 2.4.1.1 Continuously treated population

Participants in the E/E trials were pooled across treatment groups (low‐ and high‐dose groups) and were included if they were part of the E/E PET substudy, were considered amyloid positive at E/E baseline (> 20.2 CL), and had a PET scan at week 78 and before the first treatment in the LTE. Participants who completed the 78‐week double‐blind period entered the optional LTE period and were continuously treated with aducanumab until week 182 or early trial termination.

##### 2.4.1.2 Treatment gap population (subset of continuously treated population)

Qualified E/E participants were offered enrollment into EMBARK after a treatment gap due to early trial termination. Pooling the TRAC and non‐TRAC groups, the average time from E/E study baseline to end of E/E was 2.3 years and the average time from E/E study baseline to EMBARK baseline was 3.7 years. The treatment gap was 0.7 to 2.5 years (median, 1.4 years) between the end of E/E and the beginning of the EMBARK baseline assessment. Pre‐gap baseline was defined as the E/E end‐of‐study clinic visit on or after week 78. The pre‐gap baseline served as the baseline for the treatment gap population.

Participants from the continuously treated population who returned for the EMBARK redosing study were included if they had a CDR‐SB assessment at the end of the study at or after week 78, returned for screening for EMBARK after the treatment gap, and received a CDR‐SB assessment at EMBARK baseline.

### 2.5 Statistical analysis

#### 2.5.1 Continuously treated population

Characteristics at E/E baseline and cumulative drug exposure by week 78 and by the end of E/E are described in Table 1. A subset of patients assigned to titration to 10 mg/kg, the therapeutic dose approved by the US Food and Drug Administration, were included in evaluation of dose exposure.^3^, ^21^

**TABLE 1:** Characteristics of participants in the continuously treated population at E/E baseline.

Characteristic	TRAC (*n* = 112)	Non‐TRAC (*n* = 338)
**Age, mean (SD), years**	72.69 (7.57)	69.7 (6.94)
**Sex, n (%)**		
Female	56 (50)	180 (53)
**Ethnicity, *n* (%)**		
Hispanic or Latino	2 (2)	7 (2)
Not Hispanic or Latino	102 (91)	312 (92)
Not reported	7 (6)	19 (6)
Unknown	1 (1)	0
***APOE *ε4 status, *n* (%)**		
Heterozygote	48 (43)	200 (59)
Homozygote	10 (9)	72 (21)
Non‐carrier	54 (48)	66 (20)
**BMI, mean (SD), kg/m^2^**	25.81 (4.04)	25.52 (4.33)
**Duration of education, mean (SD), years**	14.62 (3.58)	15.21 (3.31)
**Duration of symptom onset, median (Q1, Q3), years**	2.9 (1.72, 4.38)	2.9 (1.81, 4.02)
**Duration of diagnosis, median (Q1, Q3), years**	0.65 (0.3, 1.25)	0.73 (0.36, 1.44)
Missing, n (%)	0	0
**Use of AD symptomatic medications, *n* (%)**		
Yes	53 (47)	194 (57)
**Clinical stage, *n* (%)**		
Mild dementia	10 (9)	57 (17)
Prodromal (MCI)	102 (91)	281 (83)
**RBANS delayed memory score, mean (SD)**	64.08 (12.79)	60.34 (13.81)
Missing, n (%)	1 (0.9)	1 (0.3)
**CDR‐GS, *n* (%)**		
0.5	112 (100)	338 (100)
**CDR‐SB, mean (SD)**	2.37 (1.03)	2.47 (0.98)
**MMSE, mean (SD)**	26.57 (1.69)	26.3 (1.82)
**ADAS‐Cog13, mean (SD)**	21.12 (6.3)	22 (6.44)
Missing, n (%)	0	1 (0.3)
**ADCS‐ADL‐MCI, mean (SD)**	43.1 (5.56)	43.32 (5.01)
Missing, n (%)	0	1 (0.3)
**NPI10, median (Q1, Q3)**	2 (0, 6.25)	2 (0, 6)
Missing, n (%)	0	4 (1.2)
**Amyloid PET CL, median (Q1, Q3)**	67.02 (45.61, 86.2)	96.54 (75.35, 122.9)
**Plasma p‐tau181, mean (SD)**	3.06 (1.31)	3.25 (1.36)
Missing, n (%)	9 (8.0)	37 (10.9)
**Whole‐brain volume, mean (SD), cm^3^**	1085.78 (109.09)	1089.37 (108.9)
Missing, n (%)	6 (5.4)	9 (2.7)
**Hippocampus volume, mean (SD), cm^3^**	4.3 (0.66)	4.15 (0.66)
Missing, n (%)	7 (6.3)	25 (7.4)

*Notes*: Characteristics at E/E baseline in the continuously treated population stratified by those who achieved versus did not achieve TRAC at week 78.

Abbreviations: AD, Alzheimer's disease; ADAS‐Cog13, 13‐item Alzheimer's Disease Assessment Scale Cognitive subscale; ADCS‐ADL‐MCI, Alzheimer's Disease Cooperative Study–Activities of Daily Living–Mild Cognitive Impairment; *APOE*, apolipoprotein E; BMI, body mass index; CDR‐GS, Clinical Dementia Rating Global Scale; CDR‐SB, Clinical Dementia Rating Sum of Boxes; CL, Centiloid; E/E, EMERGE/ENGAGE; MCI, mild cognitive impairment; MMSE, Mini‐Mental State Examination; NPI10, Neuropsychiatric Inventory 10; PET, positron emission tomography; p‐tau, phosphorylated tau; Q, quartile; RBANS, Repeatable Battery for the Assessment of Neuropsychological Status; SD, standard deviation; TRAC, treatment‐related amyloid clearance.

Participant‐level amyloid PET CL during the E/E trials was compared between those who achieved TRAC at week 78 and those who did not.

The analysis was restricted up to week 134 due to limited sample sizes at weeks 162 and 182. Mean changes from baseline in clinical outcomes (CDR‐SB, ADAS‐Cog13, ADCS‐ADL‐MCI, and MMSE) were stratified by TRAC status. Outcomes through the E/E double‐blind period and LTE were compared in participants who achieved TRAC at week 78 and those who did not. Because of the relationship between lower baseline amyloid PET and later amyloid PET levels,^22^ TRAC was expected to be more likely in participants who had a lower level of brain amyloid at baseline. Therefore, a sensitivity analysis was conducted in which participants with baseline amyloid PET < 40 CL were excluded.

Individual‐level CDR‐SB trajectories between E/E baseline and week 134 were explored by TRAC versus non‐TRAC status at week 78. The annualized CDR‐SB change was calculated between E/E baseline and week 134.

#### 2.5.2 Treatment gap population

Pre‐gap baseline (end of E/E) characteristics are described in Table 2.

**TABLE 2:** Characteristics of participants in the treatment gap population at pre‐gap baseline (end of E/E).

Characteristic	TRAC (*n* = 68)	Non‐TRAC (*n* = 196)
**Age, mean (SD), years**	75.27 (7.65)	71.66 (6.88)
***APOE *ε4 status, *n* (%)**		
Heterozygote	32 (47)	112 (57)
Homozygote	4 (6)	42 (21)
Non‐carrier	32 (47)	42 (21)
**CDR‐GS, *n* (%)**		
0	1 (1)	1 (1)
0.5	48 (71)	126 (64)
1	16 (24)	59 (30)
2	3 (4)	9 (5)
Missing	0	1 (0.5)
**CDR‐SB, median (Q1, Q3)**	3.25 (2.5, 4.5)	3.5 (2.5, 5)
**CDR‐SB, annualized change, median (Q1, Q3)**	0.31 (0, 0.81)	0.41 (0, 0.9)
**ADAS‐Cog13, median (Q1, Q3)**	24 (17.84, 28.83)	25.67 (20.33, 32.16)
Missing, n (%)	5 (7.4)	5 (2.6)
**ADAS‐Cog13, annualized change, median (Q1, Q3)**	0.95 (−0.67, 2.74)	1.89 (0.33, 3.89)
Missing, n (%)	5 (7.4)	6 (3.1)
**ADCS‐ADL‐MCI, median (Q1, Q3)**	41 (36.75, 45)	42 (37, 46)
Missing, n (%)	4 (5.9)	5 (2.6)
**ADCS‐ADL‐MCI, annualized change, median (Q1, Q3)**	−1.09 (−3.02, 0.44)	−0.82 (−2.4, 0.4)
Missing, n (%)	4 (5.9)	6 (3.1)
**Amyloid PET CL, median (Q1, Q3)**	0.16 (−7.16, 6.67)	47.45 (30.48, 62.23)
Missing, n (%)	27 (39.7)	77 (39.3)
**Amyloid PET CL, annualized change, median (Q1, Q3)**	−28.11 (−40.6, −22.2)	−21.48 (−34.53, −13.19)
Missing, n (%)	27 (39.7)	77 (39.3)
**Whole brain volume, median (Q1, Q3), cm^3^**	1026.01 (972.31, 1105.63)	1032.7 (976.6, 1122.55)
Missing, n (%)	20 (29.4)	71 (36.2)
**Whole‐brain volume, annualized change, median (Q1, Q3), cm^3^**	−12.82 (−17.01, −7.69)	−16.75 (−22.61, −10.95)
Missing, n (%)	22 (32.4)	76 (38.8)
**Hippocampus volume, median (Q1, Q3), cm^3^**	3.98 (3.41, 4.51)	3.71 (3.3, 4.28)
Missing, n (%)	19 (27.9)	77 (39.3)
**Hippocampus volume, annualized change, median (Q1, Q3), cm^3^**	−0.15 (−0.19, −0.1)	−0.15 (−0.19, −0.1)
Missing, n (%)	24 (35.3)	86 (43.9)
**ARIA‐E, n (%)**	22 (32)	64 (33)
**ARIA‐H, n (%)**	13 (19)	52 (27)

*Notes*: Characteristics at pre‐gap baseline (end of E/E) in the treatment gap population stratified by those who did versus did not achieve TRAC at week 78. Annualized change was calculated between E/E baseline and pre‐gap baseline (end of E/E).

Abbreviations: ADAS‐Cog13, 13‐item Alzheimer's Disease Assessment Scale Cognitive subscale; ADCS‐ADL‐MCI, Alzheimer's Disease Cooperative Study–Activities of Daily Living–Mild Cognitive Impairment; *APOE*, apolipoprotein E; ARIA‐E, amyloid‐related imaging abnormalities with edema or sulcal effusion; ARIA‐H, amyloid‐related imaging abnormalities with hemosiderin deposits; CDR‐GS, Clinical Dementia Rating Global Scale; CDR‐SB, Clinical Dementia Rating Sum of Boxes; CL, Centiloid; E/E, EMERGE/ENGAGE; PET, positron emission tomography; Q, quartile; SD, standard deviation; TRAC, treatment‐related amyloid clearance.

The mean changes in outcomes (CDR‐SB, ADAS‐Cog13, ADCS‐ADL‐MCI, and MMSE) from E/E baseline to the pre‐gap and EMBARK baselines after subsequent treatment cessation were compared in participants who were TRAC at week 78 versus those who were not. We conducted a sensitivity analysis in which participants with baseline amyloid PET < 40 CL were excluded.

Individual‐level CDR‐SB trajectories during the treatment gap between the end of E/E and EMBARK baseline were explored by TRAC versus non‐TRAC status at week 78. The annualized CDR‐SB change was calculated between the end of E/E and EMBARK baseline to understand clinical outcomes after TRAC when treatment was not continued.

In the TRAC group, covariates were evaluated at E/E baseline, pre‐gap baseline, or from E/E baseline to pre‐gap baseline. The association with the annualized CDR‐SB change during the treatment gap was explored. The following factors were examined to explore potential predictors of clinical outcomes post‐TRAC: apolipoprotein E (*APOE*) ε4 (carrier vs. non‐carrier) at E/E baseline; age, CDR Global Scale, CDR‐SB, ADAS‐Cog13, ADCS‐ADL‐MCI, whole‐brain volume, and hippocampus volume at pre‐gap baseline; and amyloid‐related imaging abnormalities with edema or sulcal effusion, amyloid‐related imaging abnormalities with hemosiderin deposits, annualized changes in amyloid PET CL, CDR‐SB, ADAS‐Cog13, ADCS‐ADL‐MCI, whole‐brain volume, and hippocampus volume from E/E baseline to pre‐gap baseline.

#### 2.5.3 Statistical methods

All statistical analyses were performed using R version 4.2.2. All main analyses were stratified by TRAC status at week 78. Descriptive statistics were used to summarize the cumulative dose exposure and to characterize participants at E/E baseline and pre‐gap baseline. Changes in clinical outcomes were visualized using means and standard errors. Annualized changes in CDR‐SB were summarized for the period between E/E baseline and week 134, as well as during the treatment gap. Spearman correlation within the TRAC group was used to assess the association between pre‐gap covariates and annualized CDR‐SB change after TRAC. Covariates with a Spearman correlation > 0.3 were considered to have a moderate association.

## 3 RESULTS

### 3.1 Characteristics of participants in the continuously treated population

Figure 2 illustrates the flow of patients who participated in these analyses. The continuously treated population included 450 participants, who represented 64% of those eligible for this analysis. Of the 253 not included, some discontinued the study and some had their participation truncated by early trial termination during the E/E double‐blind period prior to week 78, with most of the remainder excluded because they did not have a week 78 amyloid PET. Table 1 breaks down the continuously treated population according to TRAC status at week 78. The TRAC group comprised 25% of participants and had lower baseline amyloid burden (median, 67.0 vs. 96.5 CL), was slightly older (mean, 72.7 vs. 69.7 years) and had a slightly higher proportion of participants with mild cognitive impairment (91% vs. 83%) than the non‐TRAC group. TRAC participants additionally had more than double the proportion of *APOE* ε4 non‐carriers (48% vs. 20%) than non‐TRAC; this is likely a direct result of the dosing regimen and study protocol amendments^3^ and may not indicate a relationship between *APOE* ε4 status and likelihood to achieve TRAC.

Treatment group assignment and cumulative dose exposure differed between TRAC and non‐TRAC groups. Table S1 in supporting information shows that a higher proportion of TRAC versus non‐TRAC participants had received high‐dose aducanumab during E/E (74% vs. 41%) among those in the continuously treated population. The TRAC group had a median cumulative aducanumab dose exposure of 130 mg/kg (interquartile range [IQR], 98, 160) during the E/E double‐blind period compared to a median of 56 mg/kg (IQR, 56, 110) in the non‐TRAC group (Table S2 in supporting information). However, in a small subset of participants who received the 10 mg/kg dose, the cumulative dose by week 78 was only slightly higher in the TRAC group (*n* = 22; median, 156 mg/kg; IQR, 116, 160) than in the non‐TRAC group (*n* = 45; median, 146 mg/kg; IQR, 120, 160; Table S3 in supporting information).

### 3.2 Relationship of TRAC status to clinical outcomes up to week 134 in the continuously treated population

Figure S1 in supporting information presents spaghetti plots of the individual trajectories of amyloid PET values, stratified by dose and by TRAC status. Few were identified as TRAC at week 26. While the non‐TRAC group (regardless of dose regimen) experienced reductions in brain amyloid over time, only a negligible number achieved TRAC status after week 78.

Figure 3 shows how groups divided by TRAC status performed on the CDR‐SB over 134 weeks. Figure S2 in supporting information shows the other three clinical outcomes. On all four measures, participants in the TRAC group had numerically less decline than the non‐TRAC group by week 78. For the CDR‐SB, the TRAC group had a mean (standard error [SE]) decline of 0.96 (0.17) compared to 1.36 (0.12) in the non‐TRAC group. At week 134, the separation was larger: The TRAC group had a decline of 1.89 (0.41) compared to 2.63 (0.28) in the non‐TRAC group. The actual values shown in the current analysis cannot be compared to the original E/E values because the study group in the current analysis remained in the E/E trials until at least week 78, and roughly one third remained in the LTE until week 134 (attrition and early trial termination in the placebo group was 47% in EMERGE and 39% in ENGAGE). Figure 4 shows a histogram of annualized CDR‐SB change at week 134 from E/E baseline. The rate of CDR‐SB decline throughout the LTE was heterogeneous across individuals that did or did not achieve TRAC. For example, 34.3% of participants in the TRAC group had an annualized decline of ≥ 1 point over 134 weeks as did 38.0% in the non‐TRAC group.

**FIGURE 3:**
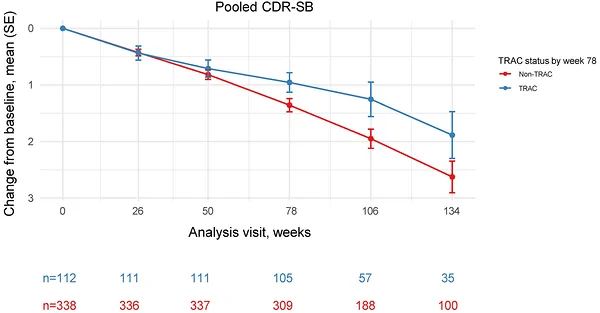
CDR‐SB change stratified by week 78 TRAC status in the pooled continuously treated population. Change in clinical outcome on the CDR‐SB, stratified by TRAC at week 78 (blue) versus non‐TRAC (red) in the continuously treated population throughout the EMERGE/ENGAGE placebo‐controlled period and long‐term extension period. Differences in inclusion and exclusion criteria used this analysis account for minor variations from the results reported by Chen et al., 2026. Adapted from Chen T, et al. *J Prev Alzheimers Dis*. 2026;13(2):100458. Creative Commons Attribution 4.0 International License (https://creativecommons.org/licenses/by/4.0/). CDR‐SB, Clinical Dementia Rating Sum of Boxes; SE, standard error; TRAC, treatment‐related amyloid clearance.

**FIGURE 4:**
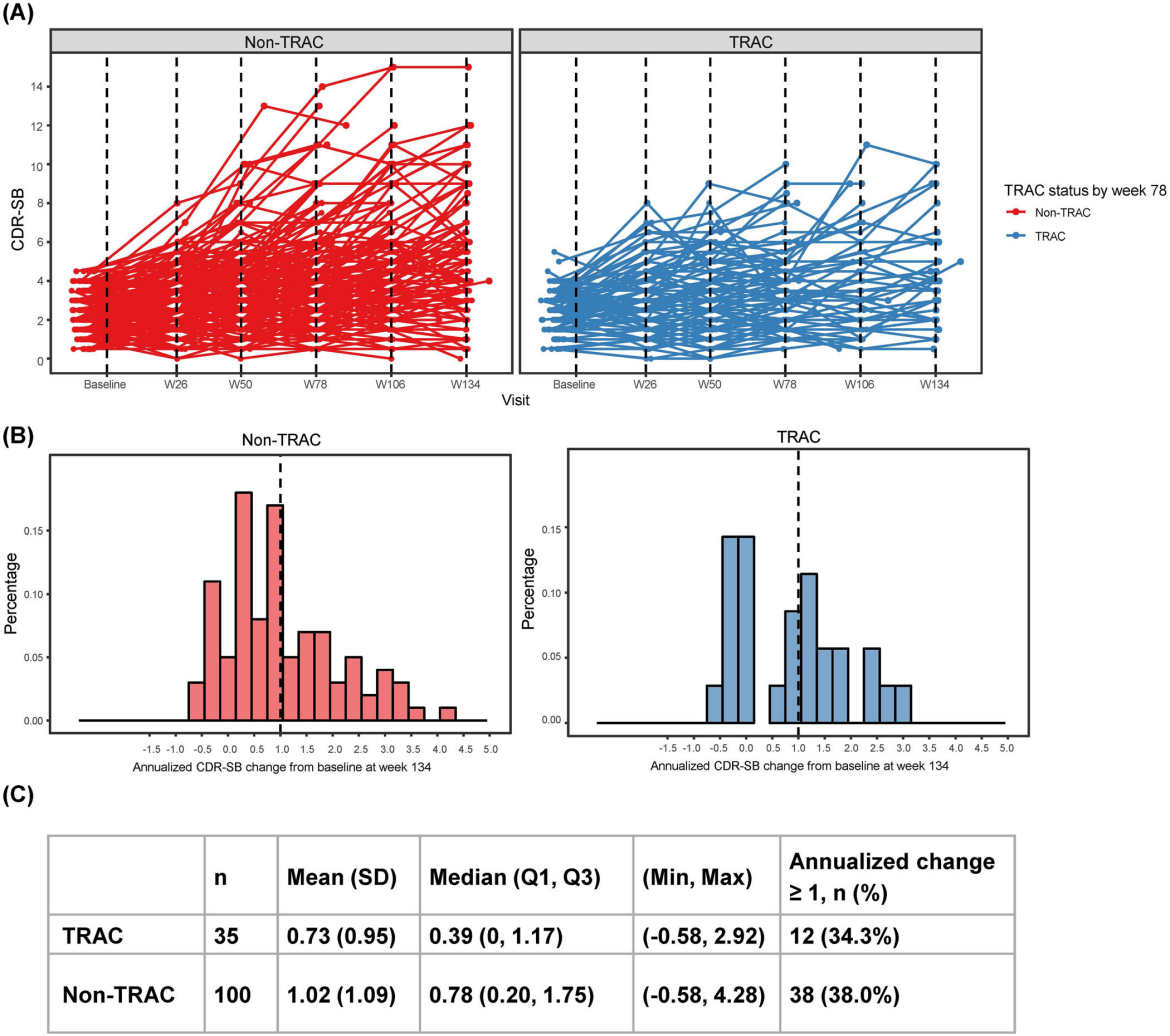
Individual CDR‐SB trajectory from E/E baseline to week 134 stratified by week 78 TRAC status in the pooled continuously treated population. A, Individual‐level CDR‐SB from E/E baseline throughout the long‐term extension to week 134 in those who achieved TRAC at week 78 (blue) versus non‐TRAC (red). B, Histogram of annualized CDR‐SB change from E/E baseline to week 134 in TRAC versus non‐TRAC groups; hashed vertical line marks an annual change of 1.0 on CDR‐SB. C, Table insert contains summary statistics of the annualized change in CDR‐SB from baseline to week 134 by week 78 TRAC status and highlights the percentage of those with a decline of ≥ 1.0 annualized points. CDR‐SB, Clinical Dementia Rating Sum of Boxes; E/E, EMERGE/ENGAGE; Q, quartile; SD, standard deviation; TRAC, treatment‐related amyloid clearance; W, week.

We conducted a sensitivity analysis to partially address the possibility of confounding by inclusion of participants who had initial lower brain amyloid levels and therefore higher probability of achieving TRAC (Figure S3 in supporting information). By excluding 26 participants whose initial amyloid PET CL value was > 20.2 but < 40, we evaluated cognitive outcomes in the subset of participants with higher baseline amyloid plaque burden. This sensitivity analysis suggests that participants with low baseline levels of brain amyloid did not attenuate or accentuate the benefits on CDR‐SB conferred by achieving TRAC status at week 78. The TRAC group had a decline (0.88 [0.18]) compared to the non‐TRAC group (1.30 [0.11]). However, this cannot fully rule out the contribution of starting amyloid levels to differences between the TRAC and non‐TRAC groups; the non‐TRAC group had more participants with higher initial amyloid PET CL (Figure S1).

### 3.3 Characteristics of participants in the treatment gap population

Figure 2 depicts the antecedent course of the 264 participants who were evaluated at the EMBARK baseline and were eligible for this analysis. The characteristics of the EMBARK participants at the end of the E/E double‐blind phase (the pre‐gap baseline) are shown in Table 2, grouped by TRAC status; 68 (26%) had achieved TRAC status at week 78. As was the case with the TRAC group in the LTE analysis, TRAC participants were more likely to be older, to be *APOE* ε4 non‐carriers, and to have less impairment at the pre‐gap time point. A higher proportion of the TRAC group in the treatment gap analysis received high‐dose aducanumab in the E/E double‐blind period compared to the non‐TRAC group (69% vs. 36%; Table S1). By design, the TRAC group's amyloid PET median value of 0.16 CL at the pre‐gap baseline was far lower than the non‐TRAC group's median level of 47.5 CL.

### 3.4 Relationship of TRAC status to clinical outcomes at EMBARK baseline in the treatment gap population

All four clinical outcomes evaluated at EMBARK baseline showed a numerically smaller decline in the TRAC group compared to the non‐TRAC group. At the EMBARK baseline (Figure 5), CDR‐SB in the TRAC group had a decline of 2.40 (0.37) compared to 3.35 (0.23) in the non‐TRAC group. The other clinical outcomes are shown in Figure S4 in supporting information. Except for the ADCS‐ADL‐MCI, separation between the TRAC and non‐TRAC groups occurred over the gap period. A sensitivity analysis (Figure S5 in supporting information) suggests that low baseline levels of brain amyloid did not appear to attenuate or accentuate the benefits on CDR‐SB. The TRAC group had a decline of 2.56 (0.41) compared to 3.32 (0.23) in the non‐TRAC group. Individual trajectories on the CDR‐SB are shown in Figure 6. A heterogeneous rate of CDR‐SB decline was shown across individuals after TRAC. During the gap period, 34% of the TRAC group had an annualized CDR‐SB decline of ≥ 1 point compared to 51% of the non‐TRAC group.

**FIGURE 5:**
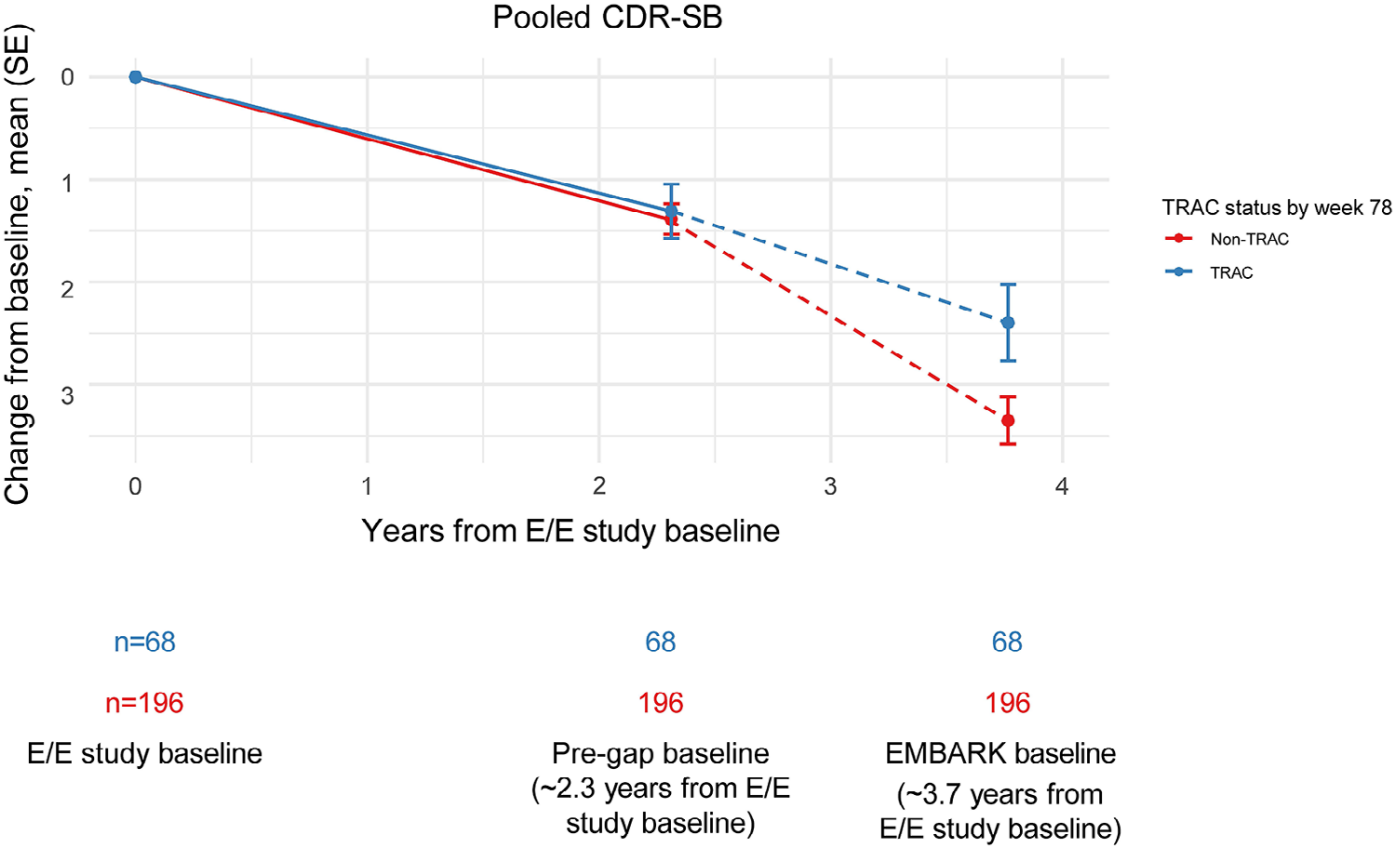
CDR‐SB change stratified by week 78 TRAC status in the pooled treatment gap population. Change in clinical outcomes on the CDR‐SB stratified by TRAC at week 78 (blue) versus non‐TRAC (red) in the treatment gap population from E/E study baseline to E/E end of study (pre‐gap baseline) and EMBARK baseline. Dashed lines mark the treatment gap over which there were no clinical measurements. CDR‐SB, Clinical Dementia Rating Sum of Boxes; E/E, EMERGE/ENGAGE; TRAC, treatment‐related amyloid clearance.

**FIGURE 6:**
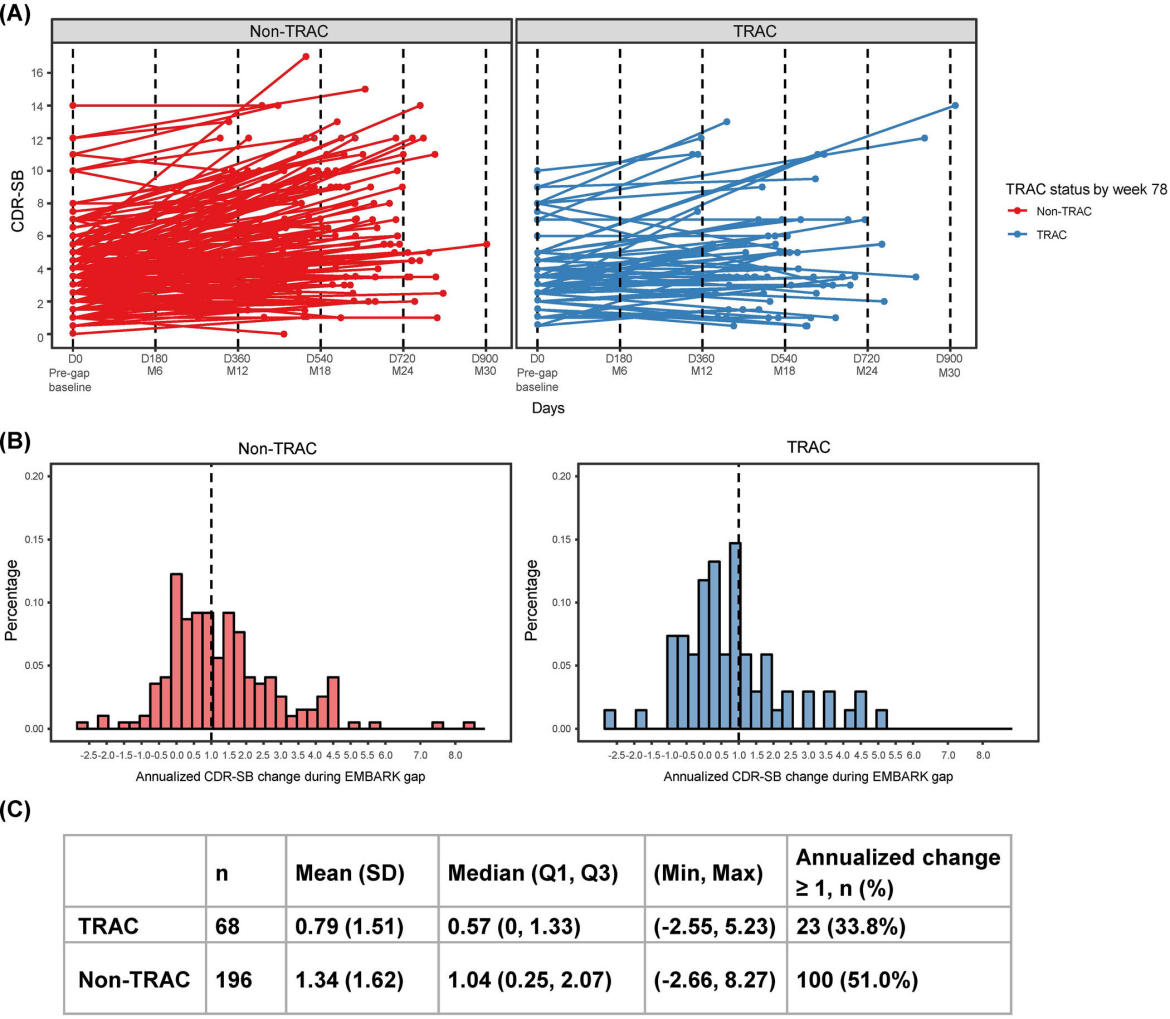
Heterogeneity in CDR‐SB during treatment gap period stratified by week 78 TRAC status in the pooled treatment gap population. A, Individual‐level CDR‐SB from the end of E/E pre‐gap baseline over the variable treatment gap to EMBARK baseline in those who achieved TRAC at week 78 (blue) versus non‐TRAC (red). B, Histogram of annualized CDR‐SB change in TRAC versus non‐TRAC during the treatment gap period; hashed vertical line marks an annual change on CDR‐SB of 1.0. C, Table insert contains summary statistics of the annualized change in CDR‐SB during the treatment gap period by Week 78 TRAC status and highlights the percentage of those with a decline ≥ 1.0 annualized point on CDR‐SB. CDR‐SB, Clinical Dementia Rating Sum of Boxes; E/E, EMERGE/ENGAGE; SD, standard deviation; TRAC, treatment‐related amyloid clearance.

### 3.5 Relationship between pre‐gap covariates and post‐TRAC disease progression after the treatment gap

Among the covariates assessed at E/E baseline, pre‐gap baseline, or from E/E baseline to pre‐gap baseline (Figure S6 in supporting information), better cognitive performance at pre‐gap baseline as measured by ADAS‐Cog13 (Spearman correlation ρ= 0.39) and greater hippocampus volume (ρ= −0.36) were associated with slower annualized CDR‐SB decline during the gap period after TRAC.

## 4 DISCUSSION

In this post hoc, exploratory analysis of post–double‐blind observations from the aducanumab phase 3 program, we focused on TRAC (defined as amyloid plaque reduction below the PET diagnostic threshold at week 78) and its impact on clinical outcomes at the end of the 78‐week double‐blind period, at week 134 after continuous treatment, and at the EMBARK baseline after a treatment gap of 0.7 to 2.5 years. Compared to non‐TRAC, the TRAC group had less clinical decline at weeks 78 and 134, and those benefits were retained a median 1.4 years later at the EMBARK baseline. Groups that achieved TRAC had greater clinical benefit than non‐TRAC when dosing after E/E was continued through week 134 (consistent with a previous report^13^) or stopped during the treatment gap. These data support consideration of TRAC as a modified disease state and underscore the importance of amyloid removal below the detection threshold as a proxy for target engagement.

Our analysis indicates that TRAC status translated into less likelihood of decline across clinical outcomes, but the degree of individual variability was considerable. We observed heterogeneity across patients in long‐term clinical outcomes after reaching TRAC in both the continuously treated and treatment gap populations, suggesting that target engagement was advantageous but not sufficient by itself consistently to affect clinical outcomes. To put this in perspective, we evaluated progression as annualized change in CDR‐SB. A 1‐point decline on this scale over 12 months has been posited as a minimal clinically important difference^23^ and clinically meaningful within‐patient change.^24^ While there was an advantage to achieving TRAC, approximately one third of TRAC participants (34.3% and 33.8% of the continuously treated and treatment gap populations) had clinically meaningful decline. Among non‐TRAC participants, 38.0% of the continuously treated and 51.0% of the treatment gap populations had a decline of ≥ 1 point on CDR‐SB. The variability with which TRAC offered clinical benefits demonstrates that TRAC status alone is not sufficient to predict future decline across individuals.

Understanding factors associated with reaching TRAC may help predict which patients are most likely to benefit from anti‐amyloid antibody treatment. TRAC was influenced by cumulative drug exposure and baseline amyloid burden. The TRAC group had nearly double the dose exposure of the non‐TRAC group. This is consistent with a previous analysis that identified sufficient cumulative dose exposure as a factor contributing to discordant results between the E/E trials;^21^ within the amyloid PET substudy, TRAC was reported in 48% of participants in the positive EMERGE study and 31% of participants in the negative ENGAGE study.^3^ In the current analysis, the TRAC group on average had 30 CL less baseline amyloid and had characteristics associated with lower baseline brain amyloid levels, such as older age, more *APOE* ε4 non‐carriers, and milder clinical syndromes.

Baseline amyloid levels have important implications for response to anti‐amyloid therapies for three reasons. First, amyloid burden generally correlates with disease severity and duration. Higher levels of extra‐medial temporal tauopathy are more likely to be seen in persons with high amyloid levels.^25^ Evidence from donanemab phase 3 studies suggested diminishing clinical responsiveness in persons with higher neocortical tauopathy.^2^ Second, lower levels of brain amyloid are more likely to be cleared compared to higher levels. Third, baseline amyloid levels set a floor on the amount of treatment‐induced removal that is possible. A previous report using the E/E dataset indicated that lower baseline amyloid PET was associated with greater percentage amyloid reduction and better clinical outcomes, particularly in those who were older and *APOE* ε4 non‐carriers.^22^ Nonetheless, the current analyses in persons receiving high‐dose aducanumab during the E/E double blind phase (see Figure S1) show that not all persons with low baseline amyloid levels achieved TRAC and that some persons with high baseline amyloid levels did achieve TRAC. This suggests that plaque removal by aducanumab operated somewhat independently of baseline amyloid levels.

Given the heterogeneity of post‐TRAC clinical outcomes, understanding factors associated with less clinical decline could help to predict which TRAC patients would be likely to progress slowly when treatment is stopped. In this analysis, better cognitive performance (ADAS‐Cog13) and higher hippocampal volume at the end of treatment were correlated with slower annualized change on CDR‐SB in the absence of ongoing treatment. Rates of post‐TRAC decline additionally may be impacted by factors not assessed here, including the presence of co‐pathology or intrinsic person‐to‐person variability in the rate of AD progression.

These analyses have several limitations, and conclusions should be interpreted within these limitations. These post hoc analyses were conducted in two longitudinal datasets from a phase 3 program in which the E/E trials were terminated early, with participants in either the double‐blind or LTE portion at the time of early termination. There was variability in cumulative dose exposure, duration of continuous exposure, and duration of the treatment gap. TRAC data were available only for participants of the longitudinal PET substudy. The definition of TRAC used here (≤ 20.2 CL at week 78) may not generalize to other settings. Participants who were above this threshold or who reached this threshold after week 78 may have experienced some benefit of treatment that cannot be observed with this approach. This definition of TRAC is specific to amyloid plaque measured by PET and does not recognize the potential effect of reduction of other amyloid species. TRAC stratification was based on post‐baseline data rather than randomization; consequently, differences in clinical outcomes could be influenced by other identified or unidentified characteristics associated with TRAC.

The exact timing of TRAC in individual participants and the rate at which amyloid plaque was reduced are unknown due to the limited number of amyloid PET assessments. Compared to achieving TRAC at the end of 78 weeks, more rapid removal of amyloid may be a plausible alternative target engagement mechanism. Unfortunately, amyloid PET imaging could not be performed with the frequency necessary to determine the rate of removal on an individual basis. Plasma biomarkers might be able to fill in the time course of amyloid removal in future analyses, but at present it is not clear whether plasma biomarkers such as phosphorylated tau (p‐tau)181 or p‐tau217 correlate well enough with therapeutic amyloid removal.^8^

Selection bias may have influenced the continuously treated population. Only 64% of initially eligible participants continued to receive treatment after week 78, with some (75/167) having withdrawn from the E/E trials prior to the end of the double‐blind period and others (92/167) stopping prematurely due to early trial termination. The withdrawals in E/E were more likely to have experienced faster decline than those who remained in the study through week 78 and/or continued into the LTE. The principal comparison in the current analysis was based on amyloid PET status at week 78, which was not known to participants or investigators, and therefore may not be subject to selection bias or unblinding.

Selection bias influenced the composition of the group that enrolled in EMBARK. Most participants who were eligible but declined to participate decided that further trial participation was not worthwhile. Participants with worse clinical decline were less likely to return to EMBARK and more likely to be in the non‐TRAC group. Thus, comparison of clinical outcomes between TRAC and non‐TRAC groups in this treatment gap population might represent a conservative estimation. Nonetheless, comparison of clinical outcomes at EMBARK baseline was based on week 78 brain amyloid levels; this is unlikely to have influenced individuals’ decisions to participate in the study.

Because these analyses were exploratory, we provide no statistical inference testing. We make no claims about divergence or convergence of the TRAC group compared to the non‐TRAC group. Instead, the key finding is a consistent quantitative benefit in the group that achieved TRAC at week 78 compared to the non‐TRAC group. Similarly, we did not assess continued or stopped dosing in two separate groups of patients with the same treatment history. Instead, the treatment gap population was a subset of the continuously treated population with varying duration of treatment exposure and treatment gap. Thus, differences between dosing scenarios are hypothesis‐generating only.

Despite these limitations, the aducanumab extended dataset offers valuable insights into the complex relationship among drug dose, baseline amyloid levels, and achieving full clearance after 78 weeks for managing the currently available anti‐amyloid antibodies. The findings of the present analysis are also consistent with reports from other programs. In a gantenerumab phase 3 exploratory analysis, a TRAC group (amyloid PET ≤ 24 CL by week 116), had numerical differences on CDR‐SB that favored treatment; no effect was observed in the non‐TRAC group and no conclusion drawn.^4^ In the donanemab program, higher baseline brain amyloid levels reduced the probability of achieving TRAC.^6^ Persons treated with donanemab who achieved TRAC (amyloid PET < 24.1 CL) had better clinical outcomes than those who did not achieve TRAC.^7^ We add to the evidence suggesting the likelihood of greater clinical benefit in those with TRAC from within‐trial^6^, ^7^ and between‐trial analyses.^5^, ^11^ The current analyses add unique information from a within‐trial comparison of aducanumab‐treated participants who did or did not achieve TRAC.

In conclusion, a benefit of TRAC at week 78 was observed on clinical outcomes in both the continuously treated cohort and the treatment gap cohort. TRAC compared to non‐TRAC demonstrated less worsening 1 year later in the continuously treated LTE and better outcomes after a 0.7‐ to 2.5‐year treatment gap prior to EMBARK. While achieving TRAC was advantageous, individual heterogeneity in post‐TRAC clinical outcomes revealed that approximately one third of TRAC patients continue to have clinically meaningful decline. These data highlight the need to further understand the benefits and limitations of amyloid plaque removal.

## CONFLICT OF INTEREST STATEMENT

Menglan Pang, Holly M. Brothers, John O'Gorman, Jennifer Murphy, Yuval Zabar, Tyler Lasky, and Ivana Rubino are employees and shareholders of Biogen. Changyu Shen was an employee of Biogen Inc. at the time of this study. David Knopman serves on a data safety monitoring board for the Dominantly Inherited Alzheimer Network Treatment Unit study sponsored by Washington University St. Louis, for the SMART‐HS clinical trial (University of Kentucky), the CRANE study (University of Michigan), and for Roche TRONTINEMAB studies WN45443 + WN45447. David Knopman was an investigator in a trial in frontotemporal degeneration with Alector. David Knopman has served as a consultant for AriBio, Biovie, and Cognito Therapeutics but receives no personal compensation. David Knopman receives funding from the NIH, the latter not related to the current work. David Knopman had previously served as a site investigator in the aducanumab program from 2015 to 2023. Author disclosures are available in the Supporting Information.

## CONSENT STATEMENT

All participants in these studies provided written informed consent.

## Supporting information



Supporting Information

Supporting Information
