# Supplementary material for: Longitudinal clinical outcomes after amyloid clearance in aducanumab phase 3 studies
Source: Alzheimers Dement. 2026 Oct 7;22(10):e71758. doi: 10.1002/alz.71758 (PMC13643829; doi:10.1002/alz.71758)
Supplement: Supplementary file 2 [file ALZ-22-e71758-s001.docx]

**Supplementary Tables and Figures**

**Supplementary Table S1. Participants With Week 78 TRAC vs Non-TRAC Status in the Analysis Populations**

| **Continuously treated population** | | | | | **Treatment gap analysis population** | |
| --- | --- | --- | --- | --- | --- | --- |
| **PET  substudy, n** | **Treatment group/dose** | | **TRAC, n**  **(n=112)** | **Non-TRAC, n**  **(n=338)** | **TRAC, n (n=68)** | **Non-TRAC, n (n=196)** |
|  | **Placebo-controlled** | **LTE** |  |  |  |  |
| 373 | High | N/A | 12 | 30 | 4 | 10 |
|  | High | High | 71 | 109 | 43 | 61 |
| 375 | Low | N/A | 7 | 33 | 3 | 20 |
|  | Low | Low | 22 | 166 | 18 | 105 |

(A) Dosing groups and number of participants who achieved TRAC at week 78 vs those who did not in the continuously treated population. (B) Number of participants who achieved TRAC at week 78 vs non-TRAC participants in the treatment gap population that entered EMBARK after an untreated, variable gap period. High dose was 6 or 10 mg/kg and low dose was 3 or 6 mg/kg, depending on study protocol version.^3, 18^ The left column represents the double-blind period, and the right column represents the LTE. N/A denotes participants who did not enter the LTE.

**Supplementary Table S2. Cumulative Aducanumab Dose Exposure by Week 78 TRAC Status in the Pooled Continuously Treated Population**

|  | **By week 78** | | **By the end of E/E** | |
| --- | --- | --- | --- | --- |
|  | **TRAC**  **(n=112)** | **Non-TRAC** **(n=338)** | **TRAC**  **(n=112)** | **Non-TRAC** **(n=338)** |
| **Mean (SD)** | 120.41 (38.21) | 82.15 (40.43) | 197.2 (88.43) | 136.38 (84.18) |
| **Median (Q1, Q3) [Min, Max]** | 130 (98, 160)  [26, 160] | 56 (56, 110)  [2, 160] | 204.5 (133, 267) [26, 400] | 108.5 (71, 202.75) [2, 390] |

Cumulative aducanumab exposure (mg/kg) in E/E at week 78, and at the end of E/E, a time point that varied per patient depending on whether they entered the long-term extension and early termination of the E/E studies.

**Supplementary Table S3. Cumulative Aducanumab Dose Exposure in Participants Receiving 10-mg/kg Dose by Week 78 TRAC Status in the Pooled Continuously Treated Population Among the Post-PV4 Participants**

|  | **By week 78** | | **By the end of E/E** | |
| --- | --- | --- | --- | --- |
|  | **TRAC**  **(n=22)** | **Non-TRAC** **(n=45)** | **TRAC**  **(n=22)** | **Non-TRAC** **(n=45)** |
| Mean (SD) | 137.82 (31.45) | 129.36 (37.6) | 159.73 (45.68) | 153.98 (54.67) |
| Median (Q1, Q3) [Min, Max] | 156.5 (115.75, 160) [47, 160] | 146 (120, 160)  [14, 160] | 170 (132.5, 190) [47, 223] | 160 (128, 200)  [14, 250] |

Cumulative aducanumab exposure (mg/kg) in participants who received the highest dose in E/E at week 78, and at the end of E/E, a time point that varied per patient depending on whether they entered the long-term extension and early termination of the E/E studies. Post-PV4 participants consented to protocol version 4^3, 18^ or later on or prior to week 16; therefore, all high-dose participants, including *APOE* ε4 carriers, were assigned to titration to the therapeutic dose of 10 mg/kg.

**Supplementary Figure S1. Individual Patient Amyloid Trajectories in the Continuously Treated Population Stratified by Week 78 TRAC Status**

Individual-level amyloid PET above (gray) and below (green) the detection threshold of 20.2 CL is plotted across time in EMERGE/ENGAGE through the placebo-controlled and LTE periods. Amyloid PET imaging was conducted at baseline and weeks 26, 78, 132, and 182. TRAC in this analysis is defined as amyloid PET ≤20.2 CL at week 78, although some in the non-TRAC group crossed this threshold after week 78. Low dose was 3 or 6 mg/kg; high dose was 6 or 10 mg/kg aducanumab. High-High and Low-Low groups continued dosing into the LTE, while N/A did not enter the LTE.

Ab, antibody; BL, baseline; CL, Centiloid; LTE, long-term extension; N/A, not applicable; TRAC, treatment-related amyloid clearance; W, week.

**Supplementary Figure S2.** **Changes in** **Additional Clinical Endpoints Stratified by Week 78 TRAC Status in the Pooled Continuously Treated Population**

Changes in clinical outcomes on MMSE (A), ADAS-Cog13 (B), and ADCS-ADL-MCI (C) stratified by TRAC at week 78 (blue) vs non-TRAC (red) in the continuously treated population throughout the EMERGE/ENGAGE placebo-controlled period and long-term extension period. Differences in inclusion and exclusion criteria used this analysis account for minor variations from the results reported by Chen et al., 2026.

ADAS-Cog13, 13-item Alzheimer’s Disease Assessment Scale–Cognitive; ADCS-ADL-MCI, Alzheimer’s Disease Cooperative Study–Activities of Daily Living–Mild Cognitive Impairment; MMSE, Mini-Mental State Examination; TRAC, treatment-related amyloid clearance.

**Supplementary Figure S3. Sensitivity Analysis of CDR-SB Change Stratified by Week 78 TRAC Status and Excluding Participants With Baseline Amyloid PET <40 CL in the Pooled Continuously Treated Population**

Change in clinical outcomes on the CDR-SB stratified by TRAC at week 78 (blue) vs non-TRAC (red) and excluding those who had baseline amyloid PET <40 CL in the continuously treated population throughout the EMERGE/ENGAGE double-blind period and long-term extension period.

CDR-SB, Clinical Dementia Rating Scale-Sum of Boxes; CL, Centiloid; PET, positron emission tomography; TRAC, treatment-related amyloid clearance.

Changes in clinical outcomes on MMSE (A), ADAS-Cog13 (B), and ADCS-ADL-MCI (C) stratified by TRAC at week 78 (blue) vs non-TRAC (red) in the treatment gap population from E/E study baseline to E/E end of study (pre-gap baseline) and EMBARK baseline. E/E end of study represents the pre-gap baseline. Dashed lines mark the treatment gap over which there were no clinical measurements. Adapted from Chen T, et al. *J Prev Alzheimers Dis*. 2026;13(2):100458. Creative Commons Attribution 4.0 International License (https://creativecommons.org/licenses/by/4.0/).

ADAS-Cog13, 13-item Alzheimer’s Disease Assessment Scale–Cognitive; ADCS-ADL-MCI, Alzheimer’s Disease Cooperative Study–Activities of Daily Living–Mild Cognitive Impairment; E/E, EMERGE/ENGAGE; MMSE, Mini-Mental State Examination; TRAC, treatment-related amyloid clearance.

Change in clinical outcomes on the CDR-SB stratified by TRAC at week 78 (blue) vs non-TRAC (red) and excluding those who had baseline amyloid PET <40 CL in the treatment gap population from E/E study baseline to E/E end of study (pre-gap baseline) and EMBARK baseline. E/E end of study represents the pre-gap baseline. Dashed lines mark the treatment gap over which there were no clinical measurements.

CDR-SB, Clinical Dementia Rating Scale Sum of Boxes; CL, Centiloid; E/E, EMERGE/ENGAGE; TRAC, treatment-related amyloid clearance.

**Supplementary Figure S6. Correlation Between CDR-SB Annualized Change During the Treatment Gap and Covariates From Pre-Gap Baseline and E/E Baseline in TRAC Participants**

(A) Correlation between CDR-SB annualized change during the treatment gap period and continuous covariates measured at pre-gap baseline or from E/E baseline to pre-gap baseline among TRAC participants. (B) Boxplot of CDR-SB annualized change during treatment gap among TRAC participants by categorical covariates measured at E/E baseline, pre-gap baseline, or from E/E baseline to pre-gap baseline. The blue lines represent the fitted mean relationship between the corresponding covariates and the annualized change in CDR during the treatment gap, estimated using a simple linear regression model. The shaded bands represent the corresponding 95% confidence intervals for the estimated mean response.

ADAS-Cog13, 13-item Alzheimer’s Disease Assessment Scale–Cognitive; ADCS-ADL-MCI, Alzheimer’s Disease Cooperative Study–Activities of Daily Living–Mild Cognitive Impairment; ARIA-E, amyloid-related imaging abnormalities with edema or sulcal effusion; ARIA-H, amyloid-related imaging abnormalities with hemosiderin deposits; CDR-SB, Clinical Dementia Rating-Sum of Boxes; E/E, EMERGE/ENGAGE; MCI, mild cognitive impairment; TRAC, treatment-related amyloid clearance.
